# Supplementary material for: Spatial cycles mediated by UNC119 solubilisation maintain Src family kinases plasma membrane localisation
Source: Nat Commun. 2017 Jul 24;8:114. doi: 10.1038/s41467-017-00116-3 (PMC5524651; doi:10.1038/s41467-017-00116-3)
Supplement: Supplementary file 1 — Supplementary Information [file 41467_2017_116_MOESM1_ESM.pdf]

Title: Supplementary Information

Description: Supplementary Figures, Supplementary Tables, and Supplementary References

Title: Peer Review File

Description:

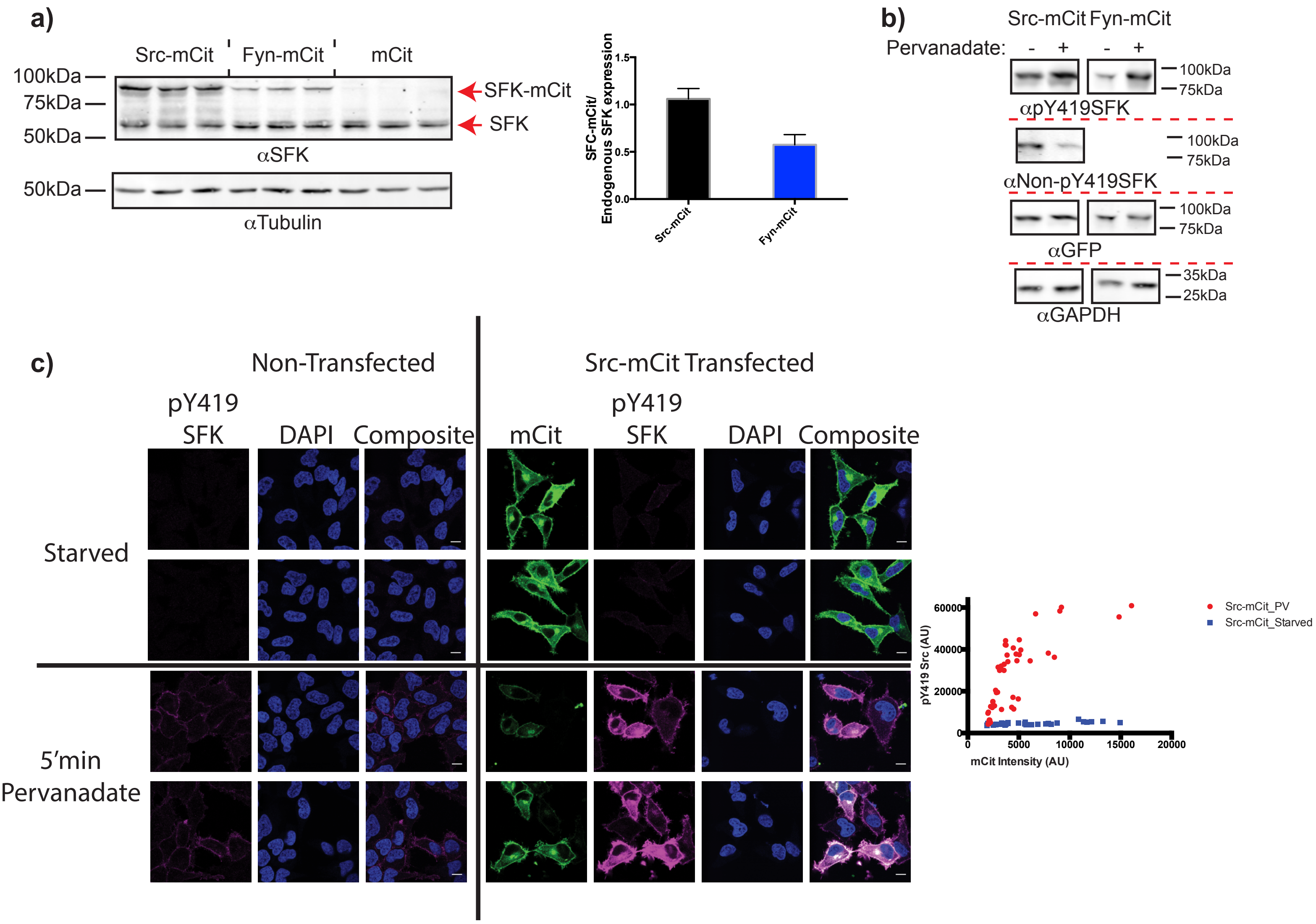

Supplementary Figure 1 **a)** HeLa cells were transfected with a vector expressing Src-mCit, Fyn-mCit or mCit alone and lysed 24hrs post-transfection. The level of expression of Src-mCit and Fyn-mCit was compared to endogenous SFK expression by probing the Western blot with an anti-SFK antibody. Each lane is from an independent repeat of the experiment on a separate day. Bar graph on the right depicts the mean ± S.D. expression of SFK-mCit, adjusted for an 80% transfection efficiency, compared to endogenous SFK levels. **b)** Representative Western blot from lysates of HeLa cells transfected with a vector expressing Src-mCit or Fyn-mCit, starved for 16hrs and then stimulated for 5min with 1mM Pervanadate or left unstimulated. SFK-mCit activation probed by Western blot with the pY419-SFK antibodies and the non-phosphorylated-Y419-SFK antibodies (n=2 independent experiments). **c)** Confocal micrographs of HeLa cells either transfected with a construct expressing Src-mCit or not transfected, starved for 16hrs and then stimulated for 5min with 1mM Pervanadate or left unstimulated. Cells were fixed, permeabilised and stained with the SFK phospho-specific antibody pY419, followed by a secondary antibody conjugated to Alexa-594. The integrated intensity of Src-mCit and the corresponding integrated intensity of the pY419 channel were then calculated for each cell and plotted to create the scatter diagram shown. Scale bars, 10 μm.

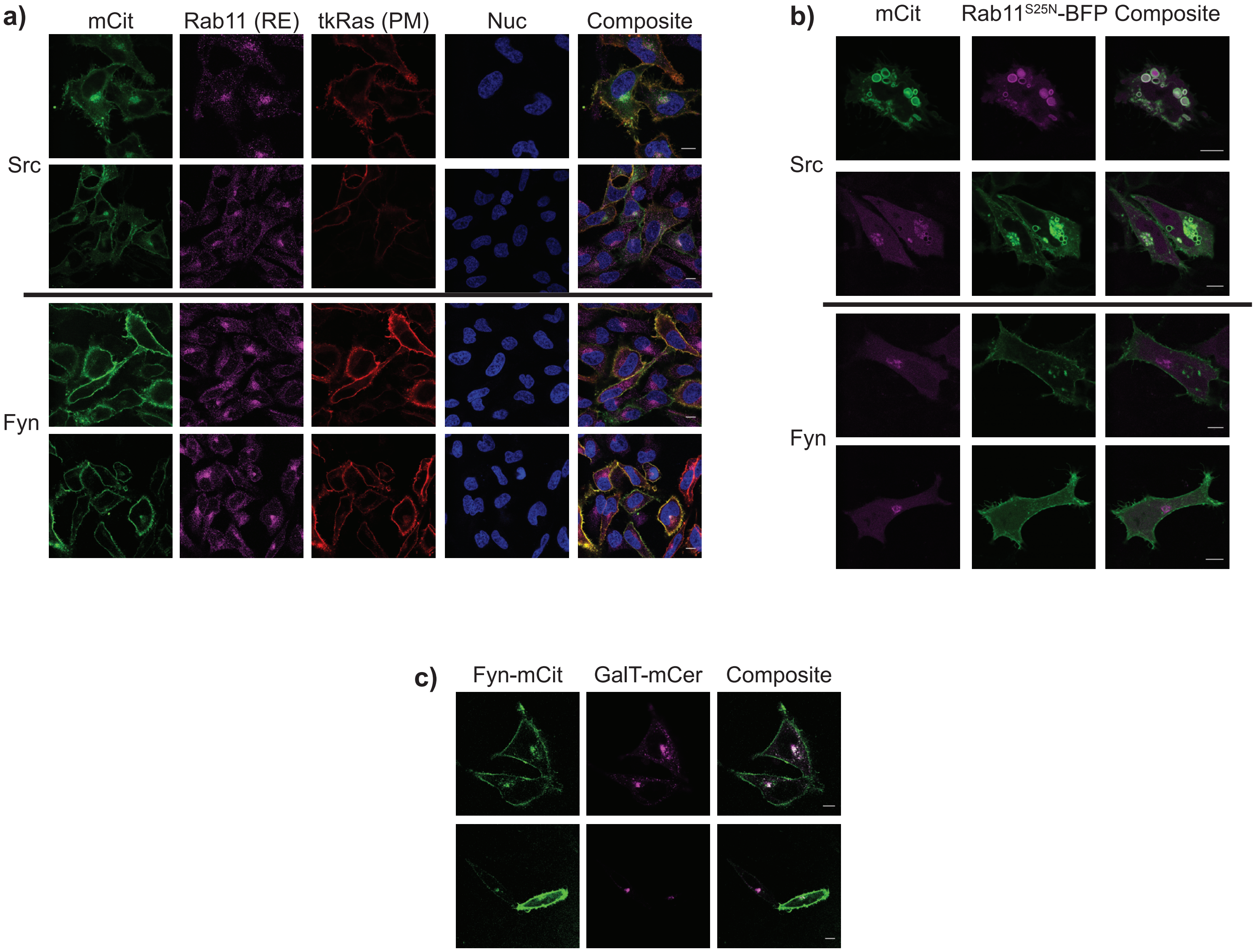

Supplementary Figure 2 **a)** Confocal images showing the steady-state localization of Src-mCit and Fyn-mCit in HeLa cells co-expressing the PM marker mCh-tK-Ras, and stained with antibodies against Rab11a. **b)** HeLa cells co-expressing the dominant negative mutant form of Rab11 (Rab11S25N-BFP) and either Src-mCit or Fyn-mCit. **c)** HeLa cells co-expressing Fyn-mCit and the Golgi marker, GalT-mCer, cultured at 20°C overnight, then treated with 50μg/ml cyclohexamide for 6hrs while still at reduced temperature. Cells were then fixed with PFA and imaged by confocal microscopy. Scale bars, 10 μm.

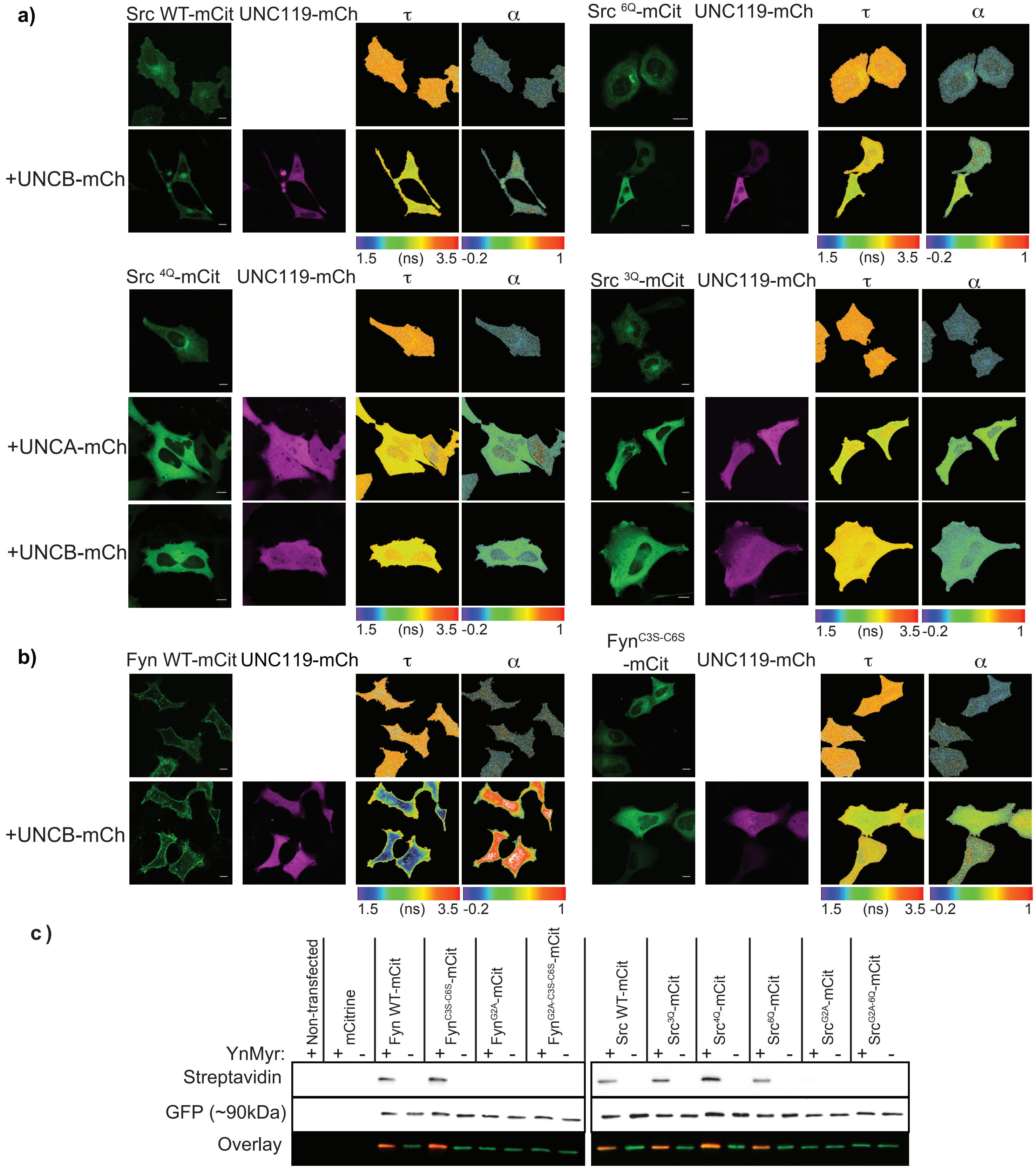

Supplementary Figure 3 FRET-FLIM of the interaction between either mCitrine fused to Src **(a)** or Fyn **(b)** and UNCA-mCh or UNCB-mCh in HeLa cells. For each sample, the fluorescence intensity of SFK-mCit (first column), the fluorescence intensity of UNC119-mCh (second column), the spatial distribution of the mean fluorescence lifetime (τ) in nanoseconds (third column) and the molar fraction of interacting molecules (α, fourth column) are shown per the false-colour look-up tables. The top rows show representative images of the donor-only samples (SFK-mCit wildtype (WT) or mutants in absence of UNC119-mCh). Images show representative measurements with Src-mCit WT and SrcnQ-mCit mutants (a) or with Fyn-mCit WT and FynC3S-C6S-mCit (b). **c)** Myristoylation levels as determined by metabolic labelling with YnMyr. HeLa cells were transfected with constructs expressing the indicated SFKs fused to mCitrine. Cells were then fed overnight with 50 µM myristic acid analog with a clickable alkyne moiety (YnMyr) or with vehicle (0.1% DMSO). Cells were lysed, and SFKs were immunoprecipitated using anti-GFP antibody. Immunoprecipitated proteins were ligated by CuAAC to Az-biotin. Tagged proteins were separated on 15% SDS-PAGE gels and analysed by IR680 labelled streptavidin fluorescence (top panel) and immunoblotting with antibody against GFP (bottom panel). Blots are representative of 3 independent experiments. Scale bars, 10 µm. See also Figure 2b and Figure 2c.

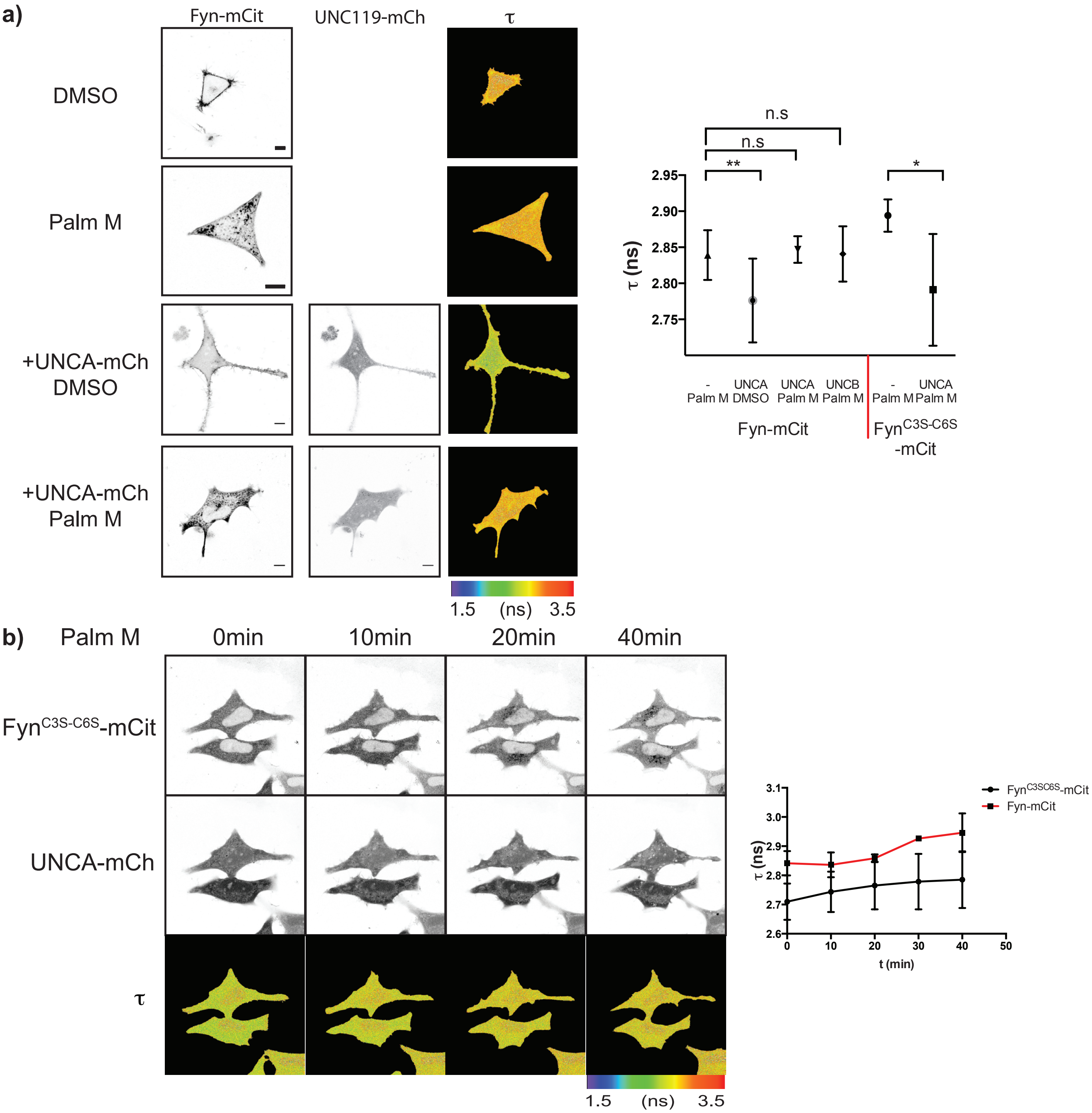

Supplementary Figure 4 **a)** FRET-FLIM measurements of the effect of thioesterase inhibition by palmostatin M (25 μM) on the interaction between Fyn-mCit and UNCA-mCh in Hela cells. For each sample, the fluorescence intensity of Fyn-mCit (donor), the fluorescence intensity of UNC119-mCh (acceptor), the spatial distribution of the mean fluorescence lifetime (τ) in nanoseconds are shown per the false-colour look-up tables. Dot plot depicts the average τ values of Fyn-mCit or FynC3S-C6S-mCit with UNCA-mCh or UNCB-mCh as the acceptor with palmostatin M (Palm M) or DMSO treatment (n=8-10 cells/condition, data are mean±S.D. from 2 independent experiments, *, P<0.05; **, P<0.01 as determined by one-way ANOVA with Bonferroni post-hoc test). **b)** FRET-FLIM time-series on HeLa cells treated with 25 μM palmostatin M to measure the effect of thioesterase inhibition on the interaction between FynC3S-C6S-mCit and UNC119A-mCh. Line graph depicts τ as a function of time after palmostatin M administration (n=10 cells for FynC3S-C6S-mCit, n=3 for Fyn-mCit, data are mean ± S.D.). Scale bars, 10 μm.

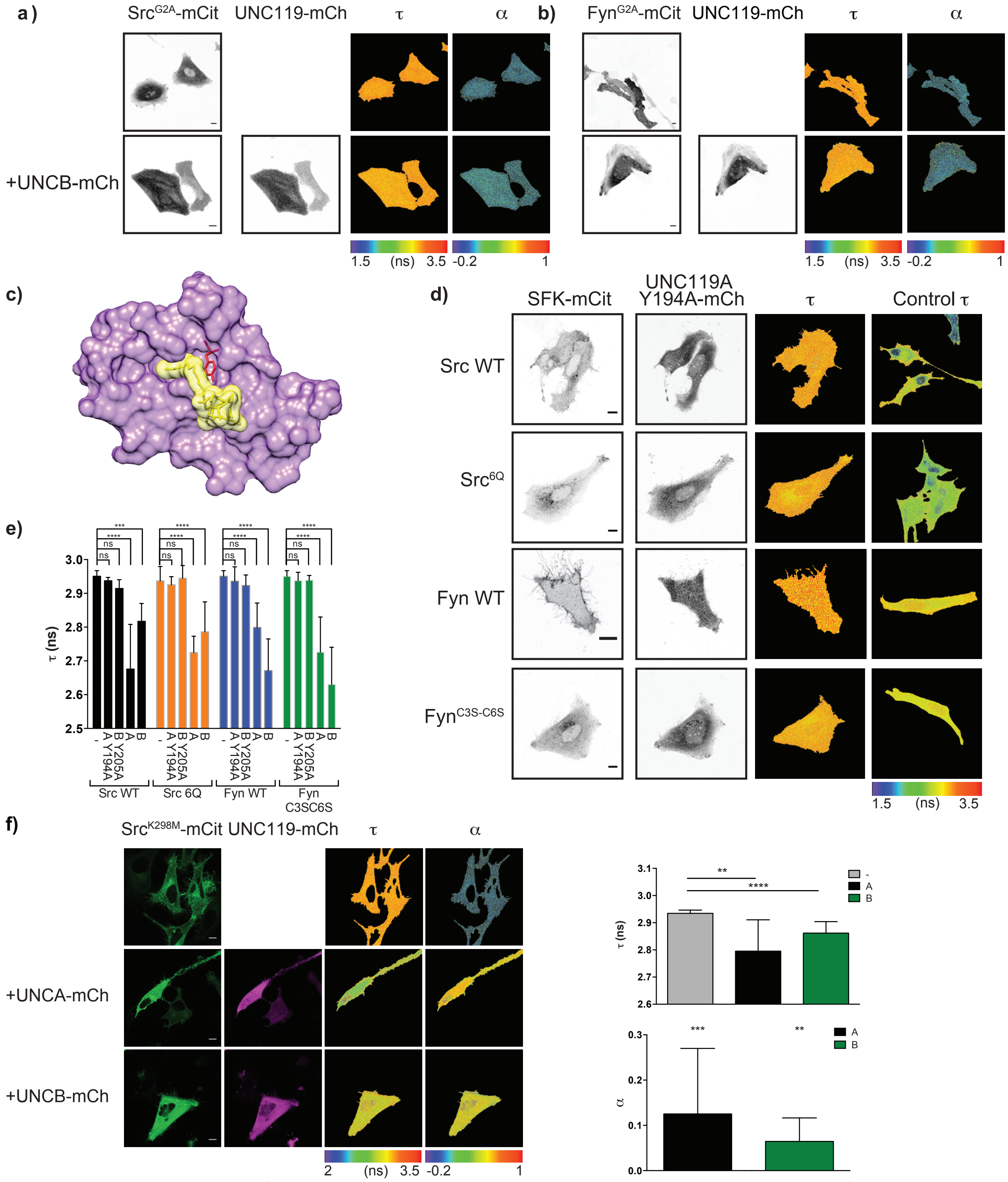

Supplementary Figure 5 FRET-FLIM of the interaction between non-myristoylatable G2A mutants of Src-mCit **(a)** or Fyn-mCit **(b)** and UNCB-mCh in HeLa cells. For each sample, the fluorescence intensity of SFK-mCit (first column), the fluorescence intensity of UNCB-mCh (second column), the spatial distribution of the mean fluorescence lifetime (τ) in nanoseconds (third column) and the molar fraction of interacting molecules (α, fourth column) are shown according to the false-colour look-up tables. The top rows show representative images of the donor-only samples (SFK-mCit mutants). **c)** Surface presentation of the lipid binding pocket of UNC119 in complex with a lauroylated GNAT-1 peptide (surface and stick presentation coloured in yellow), with Y194 shown in red (adapted from 1), depicting the contribution of Y194 to the formation of the lipid binding pocket. **d)** FRET-FLIM in HeLa cells co-expressing SFK-mCitrine (first column) with mutant UNC119Y194A-mCherry (second column). For each sample, the fluorescence intensity of SFK-mCit (donor), the fluorescence intensity of UNC119-mCh (acceptor), the spatial distribution of the mean fluorescence lifetime (τ) in nanoseconds are shown according to the false-colour look-up tables. **e)** Bar graphs depict the average τ values of the indicated SFK proteins with mutant or WT UNC119-mCh as the acceptor (n=8-10 cells /condition, data are mean±S.D. from 2 independent experiments, *, P<0.05; **, P<0.01; ***, P<0.001; ****, P<0.0001 as determined by one-way ANOVA with Bonferroni post-hoc test). **f)** FRET-FLIM measurements of the interaction between the kinase dead SrcK298M-mCit mutant and UNCA-mCh or UNCB-mCh in Hela cells. For each sample, the fluorescence intensity of SrcK298M-mCit (donor), the fluorescence intensity of UNC119-mCh (acceptor), the spatial distribution of the mean fluorescence lifetime (τ) in nanoseconds and the molar fraction of interacting molecules (α) are shown per the false-colour look-up tables. Bar graphs (right) depict average τ and α-values for SrcK298M-mCit proteins without (-), with UNCA-mCh (A), or UNCB-mCh co-expression (B) (n=24 cells/condition, data are mean±S.D. from 2 independent experiments, **, P<0.01; ***, P<0.001 as determined by one-way ANOVA with Bonferroni post-hoc test). Scale bars, 10 μm.

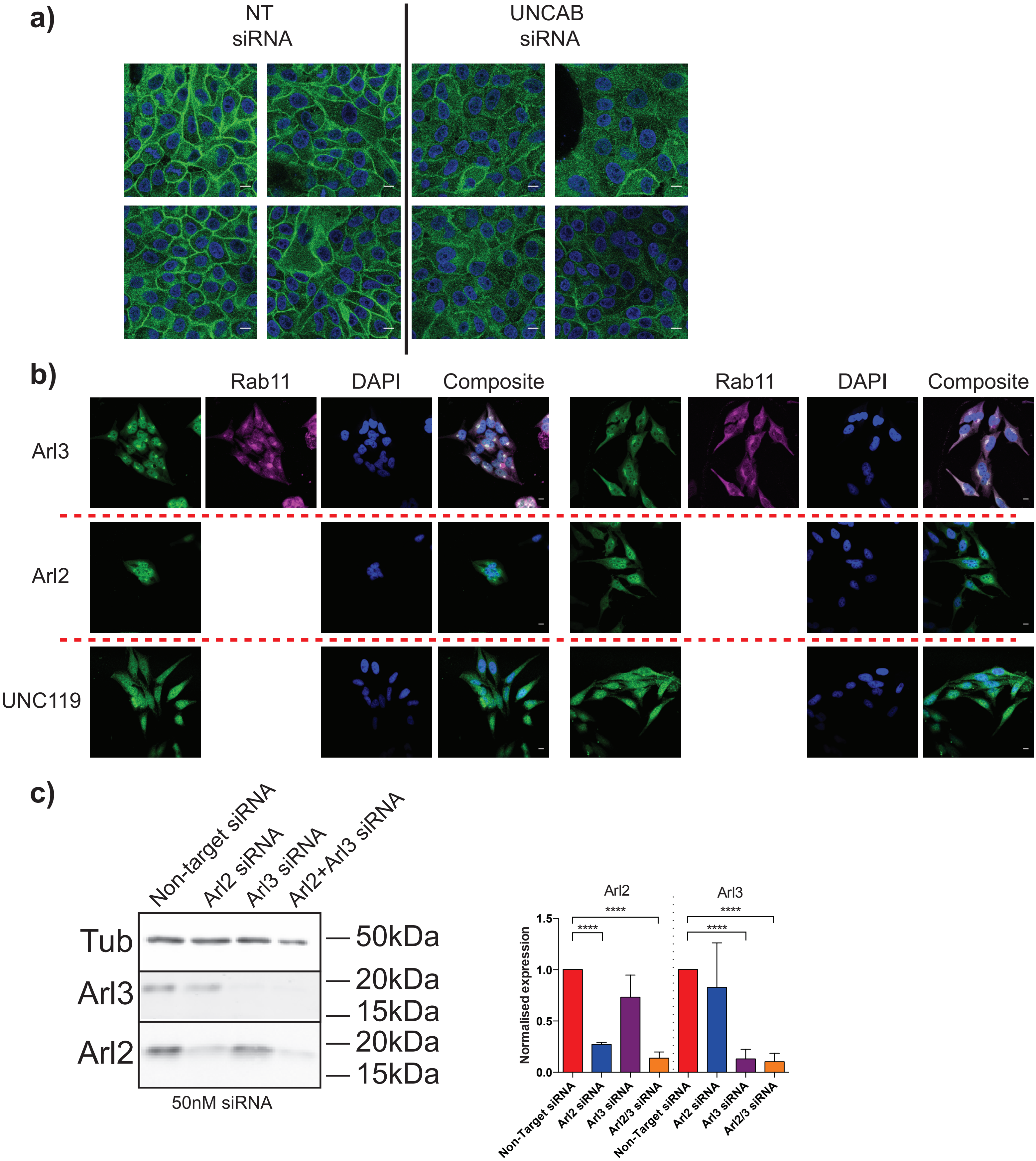

Supplementary Figure 6 **a)** Confocal micrographs of MCF10a cells transfected with UNC119A and UNC119B targeting siRNA or non-targeting siRNA control nucleotides and then stained with an antibody against SFKs (green) and with DAPI (blue). **b)** Confocal micrographs of HeLa cells stained with antibodies against Arl2, Arl3 or UNC119. Arl3 stained cells, were co-stained with antibodies against Rab11. Nuclei were stained with DAPI. **c)** HeLa cells were transfected with Arl2 and/or Arl3 siRNA and non-targeting siRNAs. 72 h post transfection cells were lysed and protein expression examined by SDS-PAGE followed by Western blotting with Arl2 and Arl3 antibodies. Blots were probed for tubulin as a loading control. Densitometry was performed on the blots; values were normalised to the tubulin loading control and compared to the non-targeting siRNA control (n=3, data are mean±S.D., ****, $P<0.0001$ as determined by one-way ANOVA with Bonferroni post-hoc test). Scale bars, 10 μm.

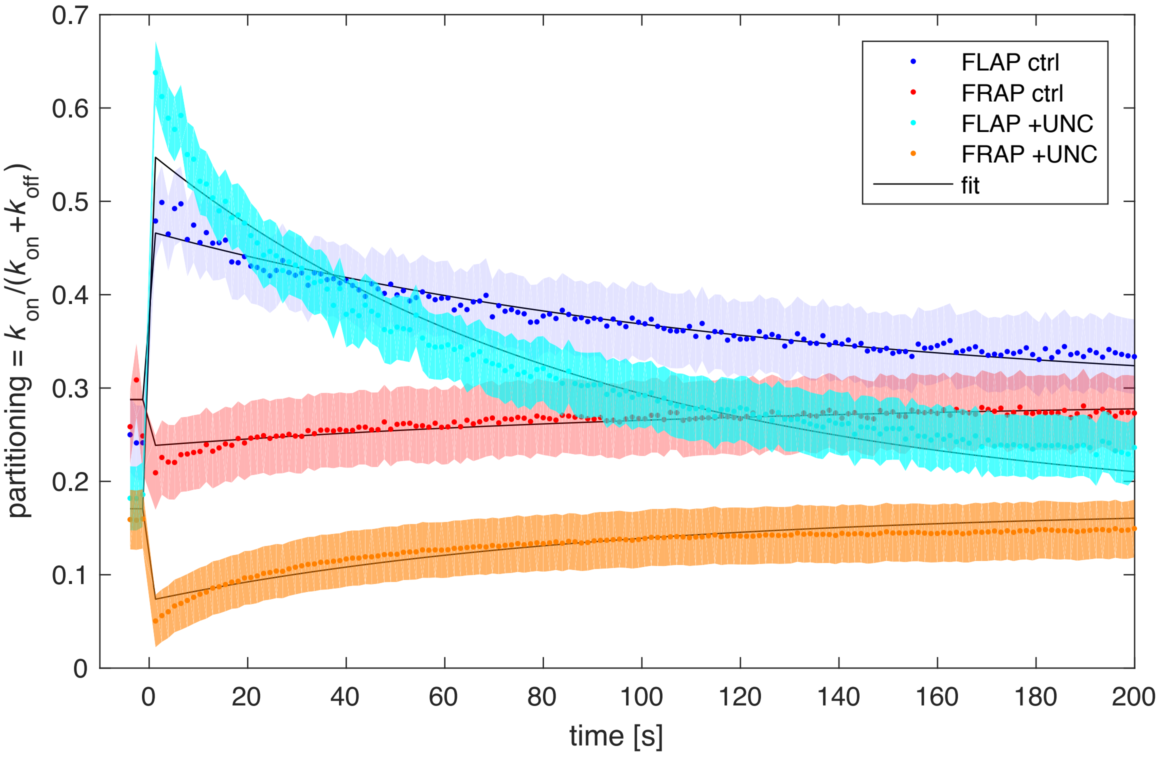

Supplementary Figure 7 Average traces of FLAP and FRAP curves as in Figure 6. The data was fitted to the compartmental model described in 'Methods', but setting the amplitude A0 of diffusion to zero. Loss of Src-paGFP fluorescence at the perinuclear region normalised to whole cell Src-paGFP fluorescence (blue and cyan curves) and corresponding gain of Src-mCh fluorescence after photobleaching at the same ROI, normalised to whole cell Src-mCh fluorescence (red and orange curves). Control cells: mean±S.E.M.; n=5 cells (blue and red curves), UNC119 ectopic expression: mean±S.E.M.; n=6 cells (cyan and orange curves).

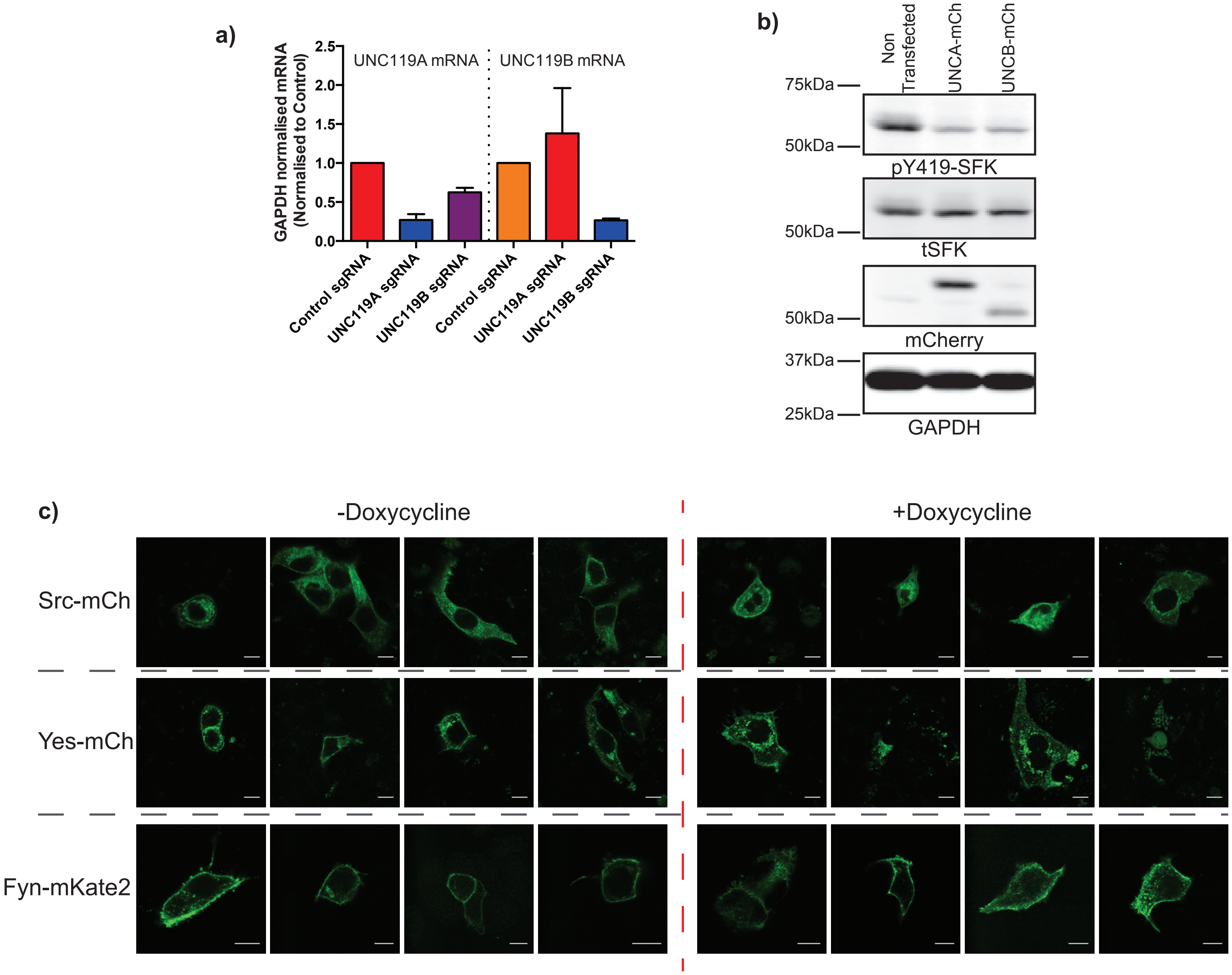

Supplementary Figure 8 **a)** UNC119A and UNC119B transcript levels were determined by qPCR on HT29 Cas9 cells 72 h after sgRNA doxycycline induction. Transcript levels were normalised to GAPDH and compared to the control sgRNA condition. **b)** Representative Western blot of HT-29 cells transfected with UNCA-mCh or UNCB-mCh constructs and lysed 48hrs post-transfection. Blots were probed with antibodies against SFK phosphorylated on Y419 (pY419-SFK), total SFK (tSFK), mCherry and GAPDH as loading control (n=3 independent experiments). **c)** Immunofluorescence micrographs of HT-29 Cas9 cells expressing the doxycycline-inducible UNC119B sgRNA and treated with or without doxycycline for 72 h, transfected with Src-mCh, Yes-mCh or Fyn-mKate2. Scale bars, 10 μm.

Insets Figure 1d

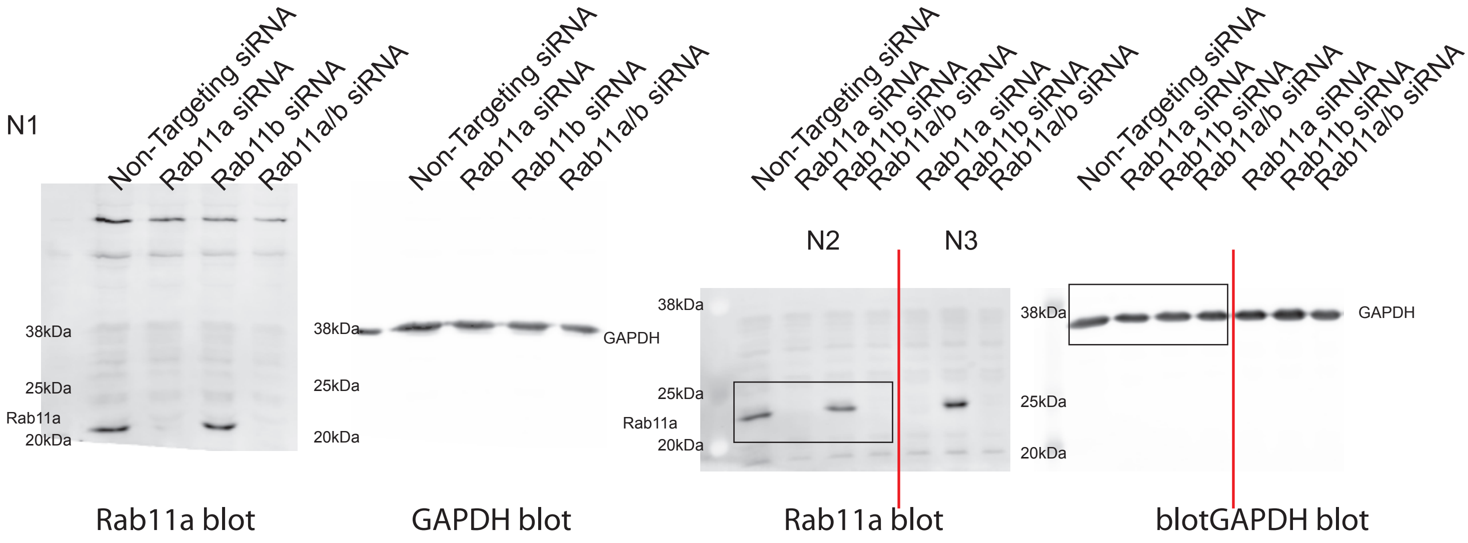

Insets Supplementary Figure 3c

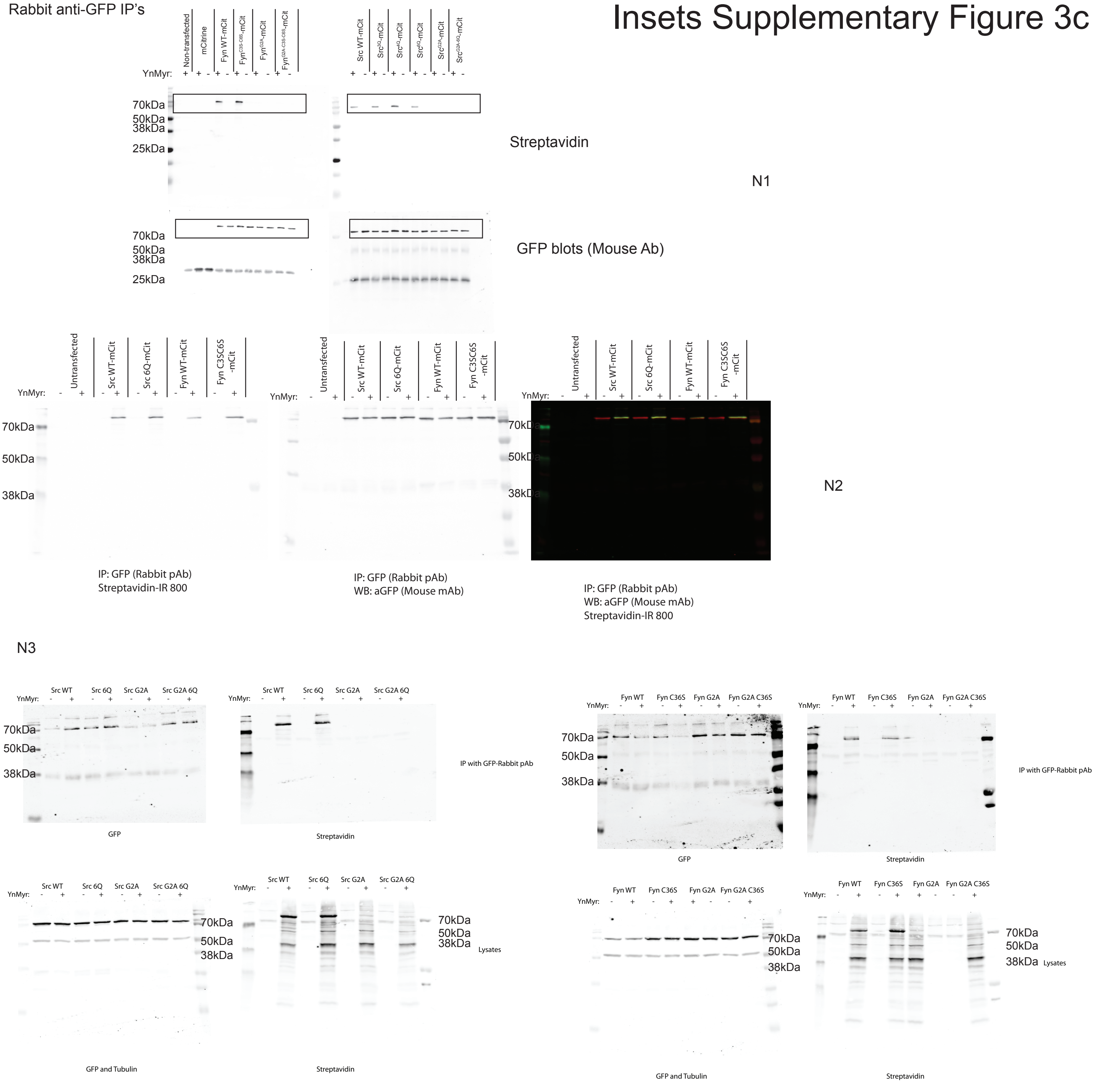

Insets and analysis Figure 3c 3d

MCF10a Cells

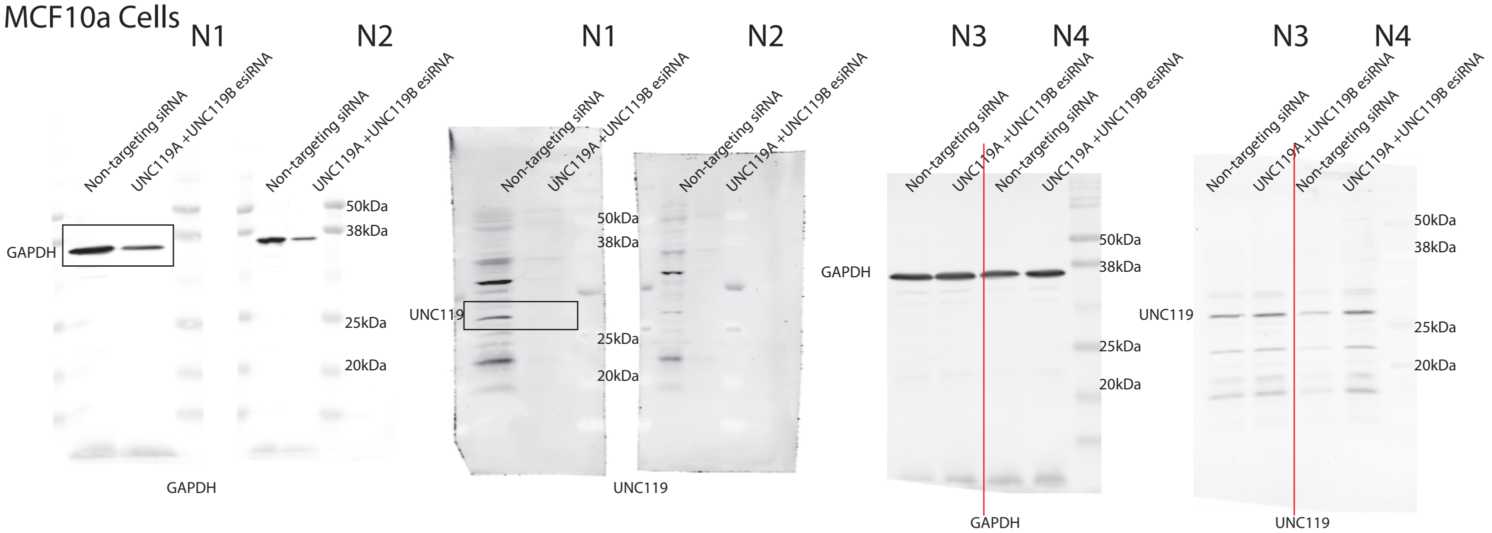

HeLa Cells

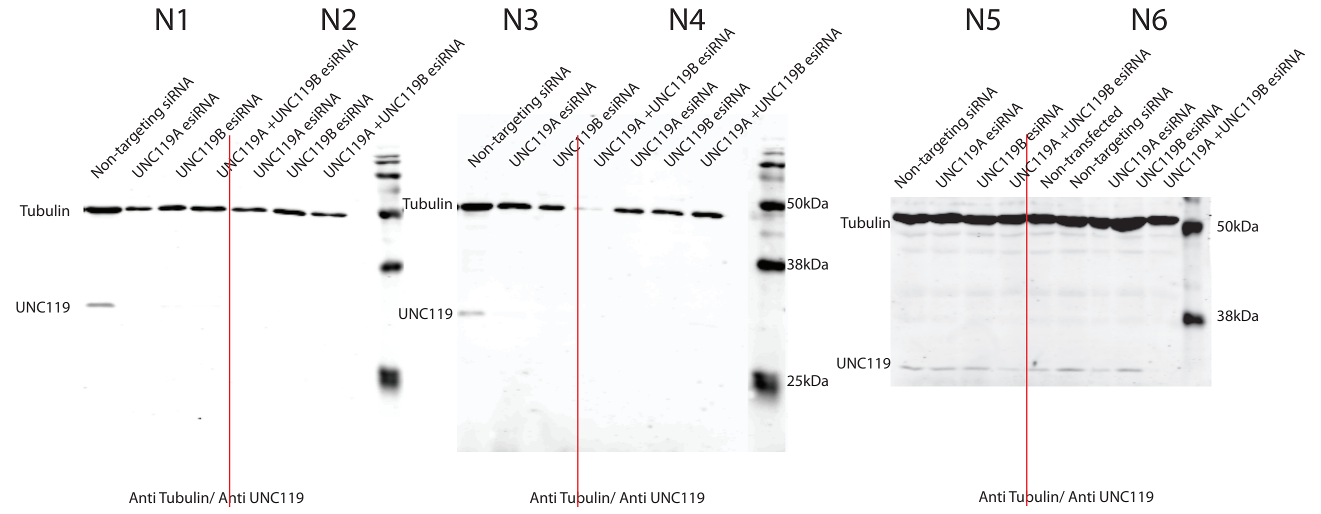

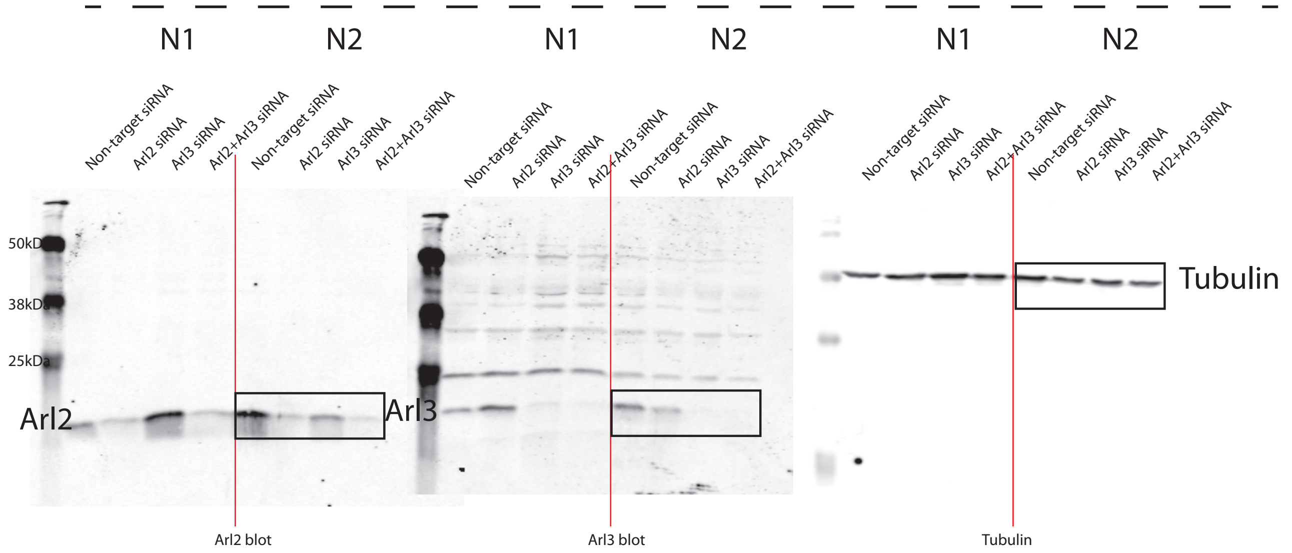

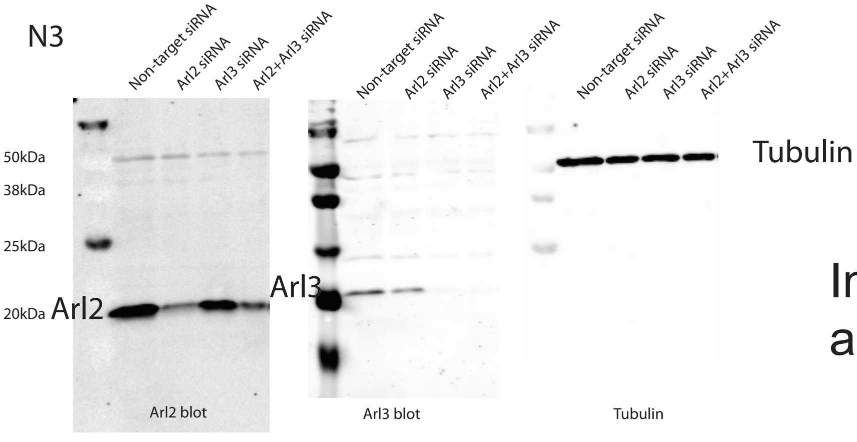

Insets and analysis Figure 6c and Figure 5

Insets and analysis Figure 7

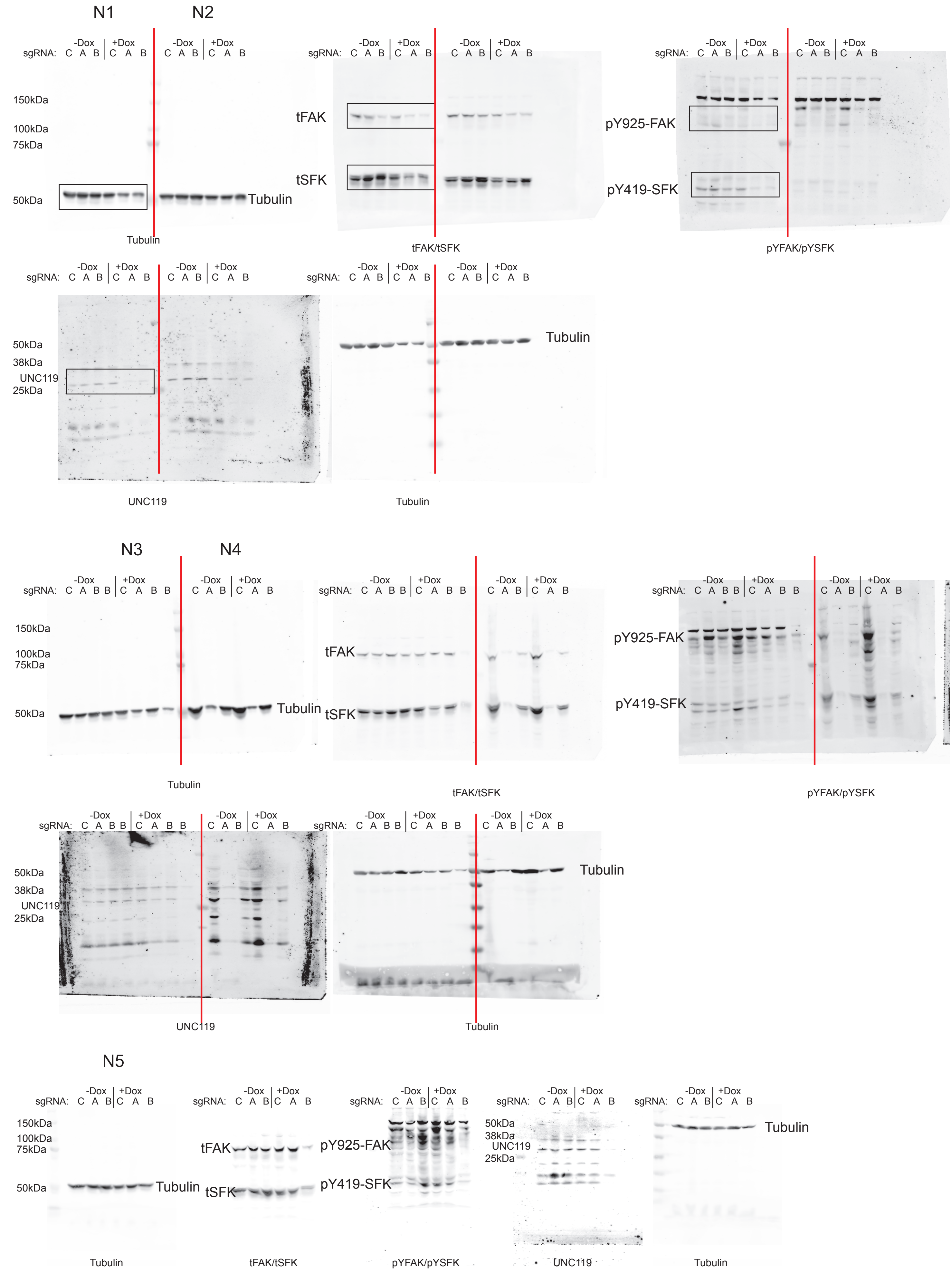

Supplementary Figure 9 Uncropped Western blots used for insets and quantitative analysis.

Supplementary Table 1. Parameters of the linked fit for the FLAP/FRAP experimental data.

Coefficients are shown with 95% confidence bounds.

	**Control**	**Ectopic tagBFP-UNC119 expression**
**Adjusted R-Squared**	0.990	0.998
$C_{0,\mathrm{FLAP}}$	0.275 (0.227, 0.322)	0.159 (0.124, 0.193)
$C_{0,\mathrm{FRAP}}$	0.221 (0.217, 0.225)	0.119 (0.109, 0.129)
D	0.77 (0.52, 1.02) μm^2 s^{-1}	1.26 (1.137, 1.382) μm^2 s^{-1}
$A_{0,\mathrm{FLAP}}$	0.24 (0.19, 0.30)	0.54 (0. 495, 0.585)
$A_{0,\mathrm{FRAP}}$	1.0 10^{-8} (fixed at bound)	0.076 (0.064, 0.088)
k_{off}	0.0175 (0.0153, 0.0197) s^{-1}	0.0297 (0.0234, 0.0354) s^{-1}
k_{on}	0.0066 (0.0058, 0.0074) s^{-1}	0.0056 (0.00449, 0.0067) s^{-1}

Supplementary References

1 Zhang, H. *et al.* UNC119 is required for G protein trafficking in sensory neurons. *Nat Neurosci* **14**, 874-880, doi:10.1038/nn.2835 (2011).
